# Unraveling the Mechanistic Basis for Control of Seed Longevity

**DOI:** 10.3390/plants14050805

**Published:** 2025-03-05

**Authors:** Shuya Tan, Jie Cao, Shichun Li, Zhonghai Li

**Affiliations:** State Key Laboratory of Tree Genetics and Breeding, College of Biological Sciences and Technology, Beijing Forestry University, Beijing 100083, China; tsy20@bjfu.edu.cn (S.T.); caojie@bjfu.edu.cn (J.C.); leeshichun99@163.com (S.L.)

**Keywords:** seed longevity, transcription factor, molecular breeding

## Abstract

Seed longevity, which holds paramount importance for agriculture and biodiversity conservation, continues to represent a formidable frontier in plant biology research. While advances have been made in identifying regulatory elements, the precise mechanisms behind seed lifespan determination remain intricate and context-specific. This comprehensive review compiles extensive findings on seed longevity across plant species, focusing on the genetic and environmental underpinnings. Inter-species differences in seed lifespan are tied to genetic traits, with numerous *Seed Longevity-Associated Gene*s (*SLAG*s) uncovered. These *SLAG*s encompass transcription factors and enzymes involved in stress responses, repair pathways, and hormone signaling. Environmental factors, particularly seed developmental conditions, significantly modulate seed longevity. Moreover, this review deliberates on the prospects of genetically engineering seed varieties with augmented longevity by precise manipulation of crucial genetic components, exemplifying the promising trajectory of seed science and its practical applications within agriculture and biodiversity preservation contexts. Collectively, our manuscript offers insights for improving seed performance and resilience in agriculture’s evolving landscape.

## 1. Introduction

Seed longevity, the inherent ability of seeds to remain viable during storage, plays a pivotal role in the perpetuation of successful plant reproduction [1]. Seed longevity varies considerably, depending on both the plant species and the employed storage conditions [1]. Gradual loss of viability over time is an inherent aspect of seed aging, driven by degradation processes that ultimately reduce seedling emergence and vigor [2]. Since seeds serve as the primary vehicle for plant propagation, maintaining seed longevity is essential not only for sustaining agricultural productivity but also for conserving plant genetic diversity [3]. Seed longevity is particularly important for cultivated crops, where it plays a vital role in ensuring high germination rates and the establishment of strong seedlings, thereby boosting crop productivity [2,3]. Given the challenges posed by climate change, including altered selection pressures and shifts in plant population genetics, seed conservation is a crucial strategy for protecting vulnerable species and plant communities that may not adapt or migrate at the same pace as environmental changes [4]. Thus, both in situ and ex situ seed conservation practices are recognized as indispensable for protecting global plant biodiversity [5]. Therefore, a thorough understanding of the complex factors influencing seed longevity carries profound ecological, agronomic, and economic implications.

Many plant species show remarkable resilience to harsh environmental conditions when their seeds are desiccated—a state characterized by substantial water loss [6]. Based on this desiccation tolerance, seeds are broadly categorized into two primary groups: recalcitrant and orthodox seeds [7]. Recalcitrant seeds are those that cannot withstand desiccation, presenting significant challenges for long-term storage, often requiring cryopreservation in liquid nitrogen. In contrast, orthodox seeds, produced by a wide range of plant species, possess desiccation tolerance and are readily stored through conventional freezing [7]. Under desiccated conditions, seeds enter a dormant phase where metabolic activity is drastically reduced, yet their potential to germinate remains intact over extended periods [8,9]. Notable examples from botanical history abound, showcasing extraordinary seed longevity: date palm seeds (*Phoenix dactylifera*) have been carbon-dated to around 2000 years old [10], sacred lotus (*Nelumbo nucifera*) seeds have retained viability after 1300 years [11], and canna (*Canna compacta*) seeds have germinated after 600 years [12]. Inspired by seminal experiments like William Beal’s seed burial test, initiated over a century ago, researchers continue to explore the mysteries of seed longevity [13], seeking answers to why certain seeds can survive for centuries longer than others [8].

Accumulating research explores the molecular underpinnings of seed longevity, investigating how specific genes might confer this exceptional durability. Studies in *Arabidopsis*, rice, barley, maize, wheat, lettuce, oilseed rape, and tobacco, among other species, have uncovered genetic determinants of seed longevity [14,15,16,17,18,19,20,21,22,23]. An extensive body of work, particularly in *Arabidopsis*, a widely used model organism, has pinpointed *Seed Longevity-Associated Gene*s (*SLAG*s) (Table 1). Manipulating these genes using molecular techniques has shown promise in altering seed longevity under experimental settings.

To systematize research efforts and facilitate further exploration, a comprehensive literature review led to the assembly of information on *SLAG*s and their corresponding mutants across multiple species. This collective endeavor resulted in the development of a dedicated database (https://ngdc.cncb.ac.cn/lsd/slag_mutant.php, accessed on 2 March 2025) [24], providing a rich resource and a solid foundation for advancing knowledge into the molecular intricacies of seed longevity. This valuable tool enables researchers to delve deeper into the mechanisms that allow certain seeds to defy time, enduring for centuries, and paves the way for targeted interventions to enhance seed survival and preserve biodiversity.

## 2. Molecular Genetics Governing Seed Longevity

### 2.1. Transcription Factors Regulating Seed Longevity

**Figure 1:**
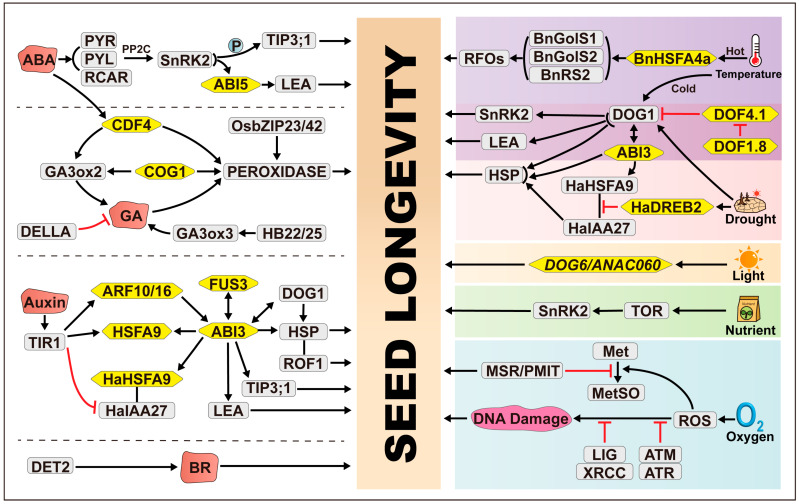
Influence of endogenous and exogenous signals on seed longevity and underlying regulatory mechanisms. Seed longevity is influenced by a variety of environmental cues, such as nutrient status, temperature, moisture, and light, as well as internal signals like plant hormones. Endogenous and exogenous stress factors have the ability to trigger diverse types of damage, such as DNA damage and protein damage, which, in turn, compromise seed longevity or vigor. Multiple plant hormones, such as ABA and GA, regulate seed longevity through modulation of transcription factors. ABA, abscisic acid; GA, gibberellic acid; BR, brassinosteroids; ROS, reactive oxygen species; PMIT, protein-L-isoaspartate (D-aspartate) O-methyltransferase; ATM, ATAXIA TELANGIECTASIA MUTATED; ATR, ATAXIA TELANGIECTASIA AND RAD3-RELATED; CDF, CYCLING DOF FACTOR; COG1, COGWHEEL1; TOR, TARGET OF RAPAMYCIN; DOF, DNA BINDING WITH ONE FINGER; DOG1, delay of germination 1; HSFA9, heat-shock factor A9; ROF1, ROTAMASE FKBP 1; HSP, heat-shock protein; TIP3;1, ALPHA-TONOPLAST INTRINSIC PROTEIN; TIR1, TRANSPORT INHIBITOR RESPONSE 1; P, phosphorylation. Yellow hexagonal shapes represent transcription factors (TFs), and irregular red shapes represent hormones. Black arrows indicate activation, while red T-shaped arrows indicate repression. Solid lines denote interactions, and double arrows denote mutual activation.

Transcriptional regulation serves as a pivotal coordinator governing diverse developmental processes and adaptive responses to a wide array of environmental challenges in plants [25,26]. At the epicenter of this regulatory mechanism are transcription factors (TFs), which are key regulatory proteins that play a vital role in virtually all aspects of plant biology. They exert their control over gene expression by interacting with specific DNA sequences (cis elements) in the promoters of their target genes or through protein–protein interactions [26,27]. Within the context of seed longevity, TFs constitute key regulators that dictate the expression of genes involved in maintaining seed vigor and viability over extended periods of storage and dormancy (Figure 1). Through their ability to bind to specific DNA sequences and modulate gene expression patterns, these TFs orchestrate a complex network that contributes to seed longevity and resilience.

Plant-specific TF ABSCISIC ACID-INSENSITIVE3 (ABI3) has emerged as a critical player in orchestrating seed dormancy and longevity via ABA-dependent pathways, as evidenced by extensive studies in *Arabidopsis* [28]. Mutations in the *ABI3* gene lead to aberrant seed maturation, compromising dormancy, desiccation tolerance, and longevity, often accompanied by impaired chlorophyll breakdown [29]. ABI3 exerts its regulatory influence by binding to the evolutionarily conserved RY motif [CATGCA(TG)] prevalent within the promoter regions of numerous seed-specific genes [30]. Notably, *Arabidopsis thaliana HEAT SHOCK TRANSCRIPTION FACTOR A9* (*AtHSFA9*) and *TONOPLAST INTRINSIC PROTEIN 3;1* (*TIP3;1*), both bearing RY motifs in their promoter sequences, serve as downstream targets of ABI3 and contribute significantly to seed longevity enhancement. While AtHSFA9 is a seed-specific heat-shock factor that bolsters longevity upon activation [31], TIP3;1, a seed-specific aquaporin, also contributes positively to longevity under ABI3 regulation [32]. Loss of function of AtHSFA2 or AtHSFA9 significantly reduces seed longevity in *Arabidopsis*, whereas overexpression of *AtHSFA2* or *AtHSFA9* leads to the increased accumulation of heat-shock proteins (HSPs) and superior seed longevity [33]. As a chaperone of HSFA2, HSP90 interacts with ROTAMASE FKBP 1 (ROF1) and ROF2 to bolster seed longevity [34] (Figure 1). Accordingly, disruption of *ROF1/ROF2* results in increased sensitivity to accelerated aging and poor germination under adverse conditions [34]. Moreover, the orthologs of *AtHSFA9* across various plant species consistently demonstrate their ability to augment seed longevity. For instance, the overexpression of *Helianthus annuus HSFA9* (*HaHSFA9*) or *Medicago truncatula HSFA9* (*MtHSFA9*) results in enhanced seed thermo-tolerance and longevity, representing promising candidates for molecular breeding interventions [35,36]. The interplay between TFs further underscores the complexity of seed longevity regulation. *Helianthus annuus DROUGHT-RESPONSIVE ELEMENT-BINDING FACTOR 2* (*HaDREB2*), an AP2/ERBP family member, amplifies the seed longevity effects of *HaHSFA9* when co-expressed, potentially by disrupting the suppressive interaction between HaHSFA9 and AUXIN-RESPONSIVE PROTEIN 27 (HaIAA27), a protein encoded by the *AUXIN/INDOLE-3-ACETIC ACID* (*Aux/IAA*) gene, thereby liberating HaHSFA9’s function to promote longevity [37,38]. However, HaDREB2 alone does not increase seed longevity without HaHSFA9.

Expanding the scope, research continues to reveal the multifaceted roles of additional TF families in regulating seed longevity. Members of the DNA BINDING WITH ONE FINGER (DOF) family, which are plant-specific TFs with a broad spectrum of biological functions [39], have been implicated in modulating seed longevity. Genetic evidence from the *Arabidopsis dof4.1* loss-of-function mutant shows enhanced seed viability following artificial aging treatments, suggesting that DOF4.1 operates as a negative regulator of seed longevity [40]. Transcriptomic analysis unveiled that the expression of *DELAY OF GERMINATION* (*DOG1*), a regulator of seed dormancy and longevity [41,42], is upregulated in the *dof4.1* mutant compared to wild-type plants, suggesting that DOF4.1 may negatively regulate seed longevity by repressing *DOG1* [40]. Conversely, COGWHEEL1 (COG1/DOF1.5) and CYCLING DOF FACTOR 4 (CDF4/DOF2.3) serve as positive regulators, with their overexpression conferring resistance to seed deterioration in Arabidopsis [43,44]. COG1 enhances seed longevity, possibly by increasing expressions of peroxidase genes based on the transcriptomic analysis of *cog1-2D*, a gain-of-function mutant with increased seed longevity [43]. Co-expression network analysis identified TFs *WRKY3* and *NFXL1* as components involved in seed longevity, as loss-of-function mutants of *wrky3* and *nflx1* exhibit reduced seed longevity [45]. A genome-wide association study (GWAS) revealed several TFs, including MYB TF (MYB47), MADS box TF (SEPALLATE 3, SPE3), and homeodomain (HB) TF (KNOTTED-LIKE HOMEOBOX OF ARABIDOPSIS THALIANA 7, KNAT7), as positive regulators of seed longevity [46]. The *athb25-1D*-dominant *Arabidopsis* mutant, with higher expression of *HOMEOBOX PROTEIN 25*, displays improved seed longevity [47], further supporting the involvement of HB TFs in seed longevity regulation.

High-throughput RNA sequencing has identified SEUSS, a transcriptional corepressor linked to embryonic development [48], as being significantly upregulated in aged seeds of *Astronium fraxinifolium* [49]. While this suggests a potential role in seed longevity, the exact regulatory mechanisms remain unclear. In the floral meristem, SEUSS is known to interact with APETALA1 (AP1) and SHORT VEGETATIVE PHASE (SVP) to repress homeotic gene expression, thereby preventing premature differentiation of the floral meristem [48]. Given this, it is plausible that, in the context of seed longevity, SEUSS may be recruited by specific transcription factors to form a complex that fine tunes the expression of genes related to seed longevity.

Seed vigor and longevity are pivotal for enhancing grain quality and germplasm conservation in crops. RNA-seq co-expression regulatory network analyses identified bZIP transcription factors *bZIP23* and *bZIP42* as candidate genes for seed longevity in rice [50]. The overexpression of *bZIP23* robustly elevates seed vigor, a process linked to the activation of *PEROXIDASE 1A* (*PER1A*), unveiling a bZIP23-PER1A-mediated detoxification pathway that fortifies seed vigor [50]. This finding underscores the potential of targeted manipulation of key TFs through genome editing as a viable strategy to boost seed vigor; overall seed quality; and, ultimately, crop yield.

In conclusion, transcriptional regulation, particularly through TFs like ABI3, DOF, MYB, MADS box, HB, and bZIP family proteins, constitutes a sophisticated network that profoundly impacts seed longevity and vigor. These discoveries provide fertile ground for innovative agricultural advancements and germplasm conservation practices in crop species. However, despite significant progress, several key gaps in our understanding of TF-mediated seed longevity remain. While individual TFs and their downstream targets have been identified, the complex interplay and combinatorial effects of multiple TFs in regulating seed longevity are still not fully elucidated. Future research should focus on dissecting the higher-order regulatory networks involving these TFs, including investigating cooperative or competitive interactions and identifying potential master regulators that coordinate the expression of multiple longevity-related genes. Furthermore, the precise mechanisms by which TFs respond to environmental cues and integrate these signals to modulate seed longevity remain largely unknown. Investigating the upstream signaling pathways that influence TF activity, as well as the post-translational modifications that might affect TF stability and function, would be valuable. Finally, while studies in *Arabidopsis* have provided a foundation for understanding seed longevity, translating these findings to crop species is crucial for practical applications. Future research should prioritize the identification and characterization of orthologous TFs in economically important crops, exploring their functional divergence and their potential for targeted manipulation through advanced breeding techniques like genome editing. A deeper understanding of the intricate regulatory networks governing seed longevity will pave the way for the development of strategies to enhance seed quality, improve crop yields, and safeguard valuable germplasm resources.

### 2.2. Impact of DNA Damage Repair on Seed Longevity

DNA damage repair is crucial for maintaining genomic integrity and ensuring the survival of organisms. Several DNA repair pathways have been identified, including homologous recombination (HR), non-homologous end joining (NHEJ), base excision repair (BER), and nucleotide excision repair (NER) [51]. HR is an accurate repair mechanism that uses a homologous sequence as a template to repair double-strand breaks (DSBs), while NHEJ directly ligates the broken DNA ends without the need for a homologous template, albeit with a higher risk of errors [51]. BER corrects small, non-helix-distorting base lesions, and NER removes bulky, helix-distorting lesions such as those caused by UV light.

Seeds in their desiccated state possess extraordinary survival capabilities, yet they face a significant challenge as substantial DNA damage accumulates during storage, accelerating seed aging and impairing vigor [52]. To unravel the intricate defense mechanisms against this damage, researchers have turned to mutants with altered DNA repair-related genes [1,53]. This line of inquiry has shed light on the essential contribution of specific elements within the DNA repair pathway to seed longevity (Figure 1).

Key players in this context include the ATAXIA TELANGIECTASIA MUTATED (ATM), ATM AND RAD3-RELATED (ATR) [54], SUPPRESSOR OF GAMMA 1 (SOG1) [55], DNA LIGASES 4 and 6 (LIG4/6) [15,56], KU70, X-RAY REPAIR CROSS COMPLEMENTING 2 (XRCC2), POLY(ADP-RIBOSE) POLYMERASE 1 (PARP1) and PARP3, EXCISION REPAIR CROSS COMPLEMENTING-GROUP 1 (ERCC1) [55], 8-OXOGUANINE (8-OXOG), and DNA GLYCOSYLASE 1 (OGG1) [57] proteins, as well as WHIRLY 1 (WHY1) and WHY3 [58].

Among the various forms of DNA damage, double-strand breaks (DSBs) are particularly detrimental [59]. The recognition of DSBs sparks intricate intracellular signaling cascades regulated by protein kinases ATM and ATR [60,61]. Mutant plants lacking functional ATM are more sensitive to DSBs and exhibit early-onset leaf senescence [62]. An intriguing observation is that seeds from ATM mutants germinate more rapidly than wild-type seeds following accelerated aging under harsh conditions of high temperature and humidity [54]. Despite this faster germination, aged ATM mutant seeds show a high prevalence of chromosomal abnormalities. Moreover, seedlings arising from these aged seeds experience reduced survival rates and slower development of true leaves compared to wild-type seedlings, emphasizing ATM’s vital role in maintaining the genomic integrity of the germinating embryo [54].

When subjected to accelerated aging, Arabidopsis mutant lines deficient in either HR (*xrcc2-1*, *why1, or why3*) or NHEJ (*ku70-1*, *ku80-3*, *or lig4 lig6*) pathways exhibit a marginally delayed germination [55,56,58]. Additionally, base excision repair (BER) and nucleotide excision repair (NER) pathways, exemplified by mutants *arp1* and *ogg1* (BER) and *ercc1* (NER), also play a role in maintaining seed viability under stress, showing slightly delayed germination under similar conditions [55]. In summary, seed longevity is critically dependent on the proper functioning of DNA repair pathways, especially those involving ATM, ATR, and other associated components. Mutations in these genes can impact seed vigor and chromosomal stability. The ATM mutants, although demonstrating accelerated germination, suffer from compromised chromosomal integrity, revealing a delicate balance between rapid germination and genomic fidelity [54]. By deepening our understanding of DNA repair mechanisms in seed aging, we can develop informed strategies to enhance seed viability and bolster crop resilience.

### 2.3. Role of Protein Repair or Homeostasis in Maintaining Seed Longevity

Reactive oxygen species (ROS) are highly reactive molecules generated during cellular metabolism, particularly under stress conditions such as desiccation and aging [63].

In seeds, ROS play a dual role: they act as signaling molecules at low concentrations but cause oxidative damage to cellular components, including proteins, lipids, and DNA, at higher levels. This oxidative damage is a hallmark of seed aging and significantly impacts seed longevity [63]. Among the cellular targets of ROS, proteins are particularly vulnerable, leading to the loss of structural integrity and functionality, which ultimately compromises seed vigor and viability.

Among the amino acids, Methionine (Met), a sulfur-containing amino acid, is notably susceptible to oxidation by ROS, transforming into methionine sulfoxide (MetSO) in its S- and R-diastereomeric forms [64,65]. This oxidation disrupts protein function and contributes to seed aging. To counteract this damage, seeds employ methionine sulfoxide reductase (MSR), an enzyme system consisting of two subtypes, MSRA and MSRB, which specifically reduce Met-S-SO and Met-R-SO, respectively, back to Met [66]. MSR activity is strongly correlated with seed longevity across various plant species [67,68,69]. For instance, in aged rice seeds, reduced MSR activity and elevated MetSO levels are associated with decreased seed vigor [67]. Overexpression of seed-specific enzyme *OsMSRB5* effectively diminishes MetSO formation, thereby enhancing seed vigor and longevity by optimizing ROS balance [67], underscoring the critical role of MSR in sustaining seed longevity.

In addition to oxidative damage, proteins inherently undergo covalent modifications during seed aging, such as the formation of abnormal isoaspartate (isoAsp) residues [70]. Protein-L-isoaspartyl methyltransferase (PIMT) plays a key role in repairing these damaged residues by catalyzing the conversion of isoAsp back to its normal aspartate form. This repair mechanism is crucial in maintaining protein functionality and seed vigor. PIMT activity, predominantly observed in seeds, has been shown to positively influence seed longevity across multiple plant species [71,72,73,74]. For example, elevated PIMT gene expression enhances seed longevity and germination vigor in Arabidopsis and chickpea [71,74]. In rice, overexpression of OsPIMT1 reduces isoAsp accumulation, improves embryo viability, and extends seed longevity. Conversely, loss of PIMT function results in decreased seed vigor under stress conditions [73], highlighting its essential role in combating the detrimental effects of isoAsp accumulation during seed aging.

Seeds can survive extreme desiccation for millennia by entering a state of quiescence [75]. This involves accumulating protective storage proteins and lipids through intricate adjustments in protein homeostasis. Recently, researchers found that disruption of proteostasis triggered by mutations of type-II metacaspase (MCA-II) proteases compromises seed longevity in *Arabidopsis* [75]. MCA-II mutant seeds fail to confine the AAA-ATPase CDC48 (CELL DIVISION CYCLE 48) to the endoplasmic reticulum, leading to the accumulation of misfolded proteins and compromised seed viability. The localization of CDC48 to the endoplasmic reticulum is contingent upon MCA-II-mediated cleavage of PUX10 (ubiquitination regulatory X domain-containing 10), an adaptor protein that regulates the association of CDC48 with lipid droplets. PUX10 cleavage facilitates the dynamic shuttling of CDC48 between lipid droplets and the endoplasmic reticulum, a critical regulatory mechanism for maintaining spatiotemporal proteolysis, lipid droplet dynamics, and overall protein homeostasis. Interestingly, removing the PUX10 adaptor in MCA-II mutant seeds partially restores proteostasis, CDC48 localization, and lipid droplet dynamics, thereby extending seed lifespan [75]. This work reveals a novel proteolytic module essential for seed longevity.

### 2.4. Role of RFOs in Regulating Seed Longevity

Raffinose family oligosaccharides (RFOs), a group of complex carbohydrates primarily found in plants, include raffinose, stachyose, and verbascose. These sugars play a critical role in seed longevity and vigor [76,77,78,79,80]. During seed maturation, RFOs accumulate alongside other compounds, such as sucrose and LEA proteins, contributing to desiccation tolerance and the preservation of cellular integrity. This, in turn, enhances seed longevity and vigor [76,77,78,79,80].

The biosynthesis of RFOs begins with the production of galactinol, a key intermediate synthesized by galactinol synthase (GolS). GolS catalyzes the transfer of a galactosyl moiety from UDP-galactose to myo-inositol, forming galactinol, which serves as the galactosyl donor for the synthesis of RFOs. During seed development, the expression of GolS is upregulated, leading to increased levels of RFOs [76]. Overexpression of *Cicer arietinum CaGolS1/2* or *Arachis duranensis AdGolS3* in *Arabidopsis* has been shown to improve seed vigor and longevity [76,81]. Additionally, mutations in *GolS* genes can lead to reduced galactinol levels and decreased seed lifespan [80]. The critical role of RFOs in longevity is further underscored by the *zmdreb2a* (*dehydration-responsive element-binding transcription factor 2a*) mutant in maize (*Zea mays*), which exhibits decreased seed longevity due to reduced expression of *ZmRS* (*raffinose synthase*), a gene responsible for raffinose synthesis, and consequently lower RFO accumulation [78]. Interestingly, the relationship between RFOs and seed vigor is complex and may vary between plant species. In *Arabidopsis*, the total RFO content and RFO/sucrose ratio, rather than individual RFO amounts, are positively correlated with seed vigor [77]. In contrast, in maize, raffinose appears to be the primary RFO associated with seed vigor [77]. The expression of *GolS* is regulated by various factors. Heat-shock cis elements (HSEs) have been identified in the promoter of *BnGolS1* in *Brassica napus* [22], suggesting regulation by heat-shock factors (HSFs). BnHSFA4a, a heat-shock transcription factor, binds to these HSEs and activates *BnGolS1* expression. Additionally, BnHSFA4a can directly regulate the expression of other genes involved in RFO biosynthesis, such as *BnGolS2* and *BnRS2*, further enhancing RFO production and improving seed longevity and stress tolerance [22].

RFOs are hydrolyzed during seed germination, but the specific genes involved in this process are not fully understood. Maize alkaline α-galactosidase 1 (ZmAGA1) is a key enzyme responsible for RFO hydrolysis [82]. Overexpression of *ZmAGA1* enhances seed germination under stress conditions but may also negatively impact seed aging tolerance [82], suggesting a potential trade-off between seed germination and longevity. In support of this observation, integrated quantitative trait locus (QTL) analyses of seed longevity in *Arabidopsis* reveal a negative correlation between seed longevity and seed dormancy [15]. In summary, RFOs play a crucial role in seed longevity and vigor. Their biosynthesis is regulated by factors such as GolS and HSFs, while the hydrolysis of RFOs during germination is mediated by enzymes like ZmAGA1. Understanding the complex interplay between RFO biosynthesis, hydrolysis, and seed quality is essential for developing strategies to improve seed longevity and vigor.

### 2.5. Hormonal Regulation of Seed Longevity

Phytohormones play a central role in orchestrating the complex series of events during seed maturation, profoundly impacting essential quality attributes such as germination potential, dormancy, and longevity [83]. Advances in molecular–genetic, biochemical, and pharmacological research have progressively uncovered the detailed contributions of phytohormones to seed longevity and the underlying regulatory mechanisms (Figure 1).

#### 2.5.1. ABA: A Central Regulator of Seed Longevity

Among the phytohormones, abscisic acid (ABA) is a pivotal regulator intricately involved in controlling seed longevity, dormancy, and desiccation tolerance [28]. Deficiencies in ABA synthesis and signaling components significantly impact seed longevity, as demonstrated in several studies [1,14,84,85]. For example, the *aba1* mutant, unable to produce epoxy-carotenoid precursors necessary for ABA biosynthesis, exhibits drastically reduced ABA levels compared to wild-type plants in *Arabidopsis*. Dominant mutations like *abi1-1* and *abi2-1*, which affect genes coding for type 2C protein phosphatases (PP2Cs), interfere with ABA signaling by inhibiting SUCROSE NON-FERMENTING 1-RELATED PROTEIN KINASE 2 (SnRK2), leading to attenuated ABA responsiveness. Notably, ABA-deficient mutants (*aba1*) and ABA-insensitive mutants (*abi1-1* and *abi2-1*) display reduced desiccation tolerance and longevity in *Arabidopsis* [86].

The perception of ABA begins with the engagement of intracellular receptors, specifically pyrabactin resistance 1 (PYR1) and PYR1-like (PYL) proteins, which form complexes with clade A PP2Cs, ultimately activating SnRK2 protein kinases in Arabidopsis [87]. Activated SnRK2s then modulate the expression of ABA-responsive genes by phosphorylating transcription factors like ABA-responsive element-binding factors (ABFs) [87]. Accordingly, mutants devoid of functional PYR/PYL, SnRK2, or ABF2/3/4 display compromised longevity relative to wild-type plants in Arabidopsis. In Arabidopsis, transcription factor ABSCISIC ACID INSENSITIVE 3 (ABI3) directly binds to the promoters of seed-specific aquaporins TIP3;1 and TIP3;2, enhancing their expression and, thus, improving seed longevity [88]. In summary, ABA plays a central role in regulating seed longevity by orchestrating desiccation tolerance, dormancy, and stress responses, making it a key target for improving seed storage and resilience in crops.

#### 2.5.2. Impact of Auxin on Seed Longevity

Auxin, a pivotal plant hormone, plays a complex role in the attainment of seed longevity [38,89,90]. During the maturation of *Arabidopsis* seeds, there is a concurrent escalation and spatial distribution of auxin signaling inputs and outputs within the embryo, tightly aligned with the seed’s journey towards achieving longevity [90]. Experimental supplementation of auxin during the maturation phase has been demonstrated to enhance seed longevity. Arabidopsis mutants with dysfunctional auxin biosynthesis pathways consistently exhibit altered longevity, reflecting a clear dose–response relationship that is tied to the intensity of auxin signaling activity [90]. The identification of a conserved gene network related to seed longevity, enriched with the cis-regulatory element TGTCTC, an auxin response factor binding site, underscores the direct link between auxin signaling and the acquisition of longevity [45]. Moreover, biochemical evidence reveals that auxins enhance seed longevity by destabilizing the *Helianthus annuus* AUXIN/INDOLE-3-ACETIC ACID 27 (HaIAA27) protein and, thus, stimulating *HSFA9* expression [38], providing additional insights into the molecular mechanisms underlying auxin regulation of seed longevity.

Auxin’s downstream actions intersect with the ABA signaling cascade within the embryo. It has been found that auxin promotes the expression of *ABI3* and its LEA protein target, *EARLY METHIONINE1* (*EM1*), with ABI3 activity shown to be dysregulated in auxin biosynthesis mutant *cyp79b2* [90]. More importantly, the positive effect of external auxin application on seed longevity during development is negated in a*bi3-1* mutants, underscoring the synergy between auxin and ABA pathways [90]. Beyond its interaction with the ABA signaling pathway, auxin may also directly modulate genes pertinent to seed longevity, implicating its involvement through both ABA-dependent and independent routes. This dual regulatory mechanism suggests that auxin plays a multifaceted role in the complex regulatory web governing seed longevity. In crop breeding, optimizing auxin signaling pathways could improve seed longevity, enhancing storage stability and viability. This may involve the use of genetic engineering to boost auxin biosynthesis or the application of auxin treatments during seed maturation, particularly in species prone to rapid seed deterioration.

#### 2.5.3. Influence of Gibberellins (GAs) on Seed Longevity

GAs are known for their prominent role in triggering seed germination and subsequent growth [91]. Comparative analyses of higher longevity (HL) and lower longevity (LL) varieties after natural aging have led to the identification of specific long-lived mRNAs in rice, including gibberellin receptor gene GID1. Seeds store various long-lived mRNAs, some of which are crucial for the early stages of germination and, consequently, for seed longevity. These findings suggest that gibberellin signaling plays a role in seed longevity [92]. Genetic analysis has shown that overexpression of *ARABIDOPSIS THALIANA HOMEOBOX 25* (*AtHB25*) in an *Arabidopsis* ‘activation tagging’ line collection resulted in elevated levels of active gibberellins and increased transcripts of gibberellin biosynthesis gene *GIBBERELLIN 3-OXIDASE 2* [47]. This increased gibberellin activity was correlated with improved resistance to controlled deterioration tests (CDTs). It is worth noting that GA3-treated plants and the quintuple DELLA mutant, characterized by persistent gibberellin responses, displayed stronger CDT resistance, pointing to a potentially positive role of gibberellins in enhancing seed longevity [47]. However, conflicting evidence arises from mutants like *ga1-3*, which is defective in gibberellin synthesis, and the gibberellin-insensitive *gai* mutant, neither of which showed decreased germination following prolonged dry storage when compared to wild-type plants [93]. This inconsistency highlights that while there is suggestive evidence for gibberellins’ participation in seed longevity, more research is needed to definitively establish their precise role.

Despite the wealth of research affirming ABA’s role in seed longevity, the contribution of other hormones remains less clear-cut [94]. Studies have shown that mutants resistant to ethylene and jasmonic acid do not significantly lose viability after long-term storage, implying a limited role for these hormones in regulating longevity [84]. On a separate note, recent findings indicate that brassinosteroids (BRs) might negatively affect seed longevity during the priming process, a controlled treatment designed to improve germination performance [95]. Seeds from BR-deficient mutants such as *cyp85a1/a2* and *det2* demonstrate prolonged longevity post priming in *Arabidopsis* [95], suggesting a possible connection between BR signaling and seed longevity, an area that merits further investigation. Modulating GA signaling pathways represents a valuable strategy for enhancing seed longevity and storage stability in crop breeding. Approaches could include overexpressing genes involved in GA biosynthesis or selecting for desirable traits associated with enhanced GA activity. Additionally, reducing BR signaling during seed priming may further extend seed longevity, offering the potential to develop crops with improved shelf life and germination performance.

### 2.6. Seed Dormancy and Longevity: Positive and Negative Correlations

Seed dormancy and longevity are both critical traits for plant survival and agricultural productivity. Ideally, a favorable correlation between these traits would benefit both natural ecosystems and crop cultivation. However, studies have reported both positive and negative correlations between seed dormancy and longevity [96].

The testa—or seed coat—is the protective outer layer of the seed that shields the embryo from adverse environmental conditions, such as mechanical damage, pathogen invasion, and desiccation. Mutations in the *TRANSPARENT TESTA* (*TT*) genes, which regulate flavonoid biosynthesis and deposition in the seed coat, decrease both seed dormancy and longevity in Arabidopsis. Flavonoids, a class of secondary metabolites, play a crucial role in seed coat integrity by contributing to its impermeability and antioxidant properties [97]. Disruptions in flavonoid deposition increase seed coat permeability, leading to greater susceptibility to environmental stressors and reduced seed longevity [96]. Disruptions in cutin biosynthesis and deposition, caused by mutations in genes such as *GPAT4/8*, *DCR*, *LACS2*, or *BDG1*, also compromise seed dormancy and longevity in *Arabidopsis* [98]. Mutations in the *VITAMIN E DEFICIENT 1* (*VTE1*) gene, which encodes a key enzyme in tocopherol-mediated antioxidant activity, also reduce both dormancy and longevity. Tocopherols protect seeds from oxidative damage during storage, and their deficiency accelerates seed aging [99]. Interestingly, a loss-of-function mutation in *RBOHD*, which encodes an NADPH oxidase, results in increased dormancy and longevity in *Arabidopsis* compared to the wild type [46,100]. Additionally, using a tetragenic system, researchers discovered that natural genes controlling seed dormancy are also involved in the regulation of soil seed bank longevity in rice [101]. Interestingly, auxin has the effect of simultaneously enhancing seed dormancy and extending lifespan by increasing ABA signaling [89,90]. These findings suggest a positive correlation between seed dormancy and longevity.

Higher-order *DELLA* (negative regulators of GA signaling) mutants in a gibberellin-deficient background (*ga1-3*) exhibit reduced dormancy due to the constitutive activation of gibberellin signaling in *Arabidopsis* [102]. Conversely, the *DELLA* quintuple mutant demonstrates increased resistance to accelerated aging, likely attributable to enhanced seed coat mucilage production [47]. Disruption of *CYP707A1/A2*, which are involved in ABA catabolism, results in enhanced dormancy but reduced longevity in *Arabidopsis* [103]. Similarly, aspartic protease ASPG1, which is responsible for seed reserve mobilization, shows increased dormancy but decreased longevity in mutants with reduced proteolytic activity [104]. Auxin biosynthesis mutants, such as *taa1*, *tar1*, and *yuc1*, display reduced dormancy but increased longevity in *Arabidopsis* [90]. Additionally, quantitative trait locus (QTL) analyses in recombinant inbred line populations have revealed a negative correlation between seed dormancy and seed longevity in *Arabidopsis* [15]. These findings suggest that ABA catabolism and auxin biosynthesis play pivotal roles in the observed negative correlation between seed dormancy and longevity.

In summary, genetic analyses have demonstrated both positive and negative correlations between seed dormancy and longevity, reflecting the complexity of their regulation. The contradictory findings regarding the relationship between seed dormancy and longevity may depend on species-specific or cultivar-specific genetic backgrounds, environmental conditions, and experimental methodologies. For instance, variations in seed coat composition, hormonal regulation, and stress response mechanisms across species could contribute to these discrepancies. Additionally, the interplay between shared and independent signaling pathways, such as those involving ABA, auxin, and gibberellins, may differentially influence dormancy and longevity in different contexts. Further research is needed to elucidate the molecular and ecological factors driving these correlations and to determine how they can be harnessed for crop improvement.

## 3. Environmental Regulation of Seed Longevity

Seed longevity is strongly influenced by a multitude of environmental factors in conjunction with genetic determinants [105,106] (Figure 1). The environment experienced by the maternal plant during seed maturation, along with the conditions following harvest and throughout storage, plays a critical role in determining seed viability [18,107]. Key environmental parameters that significantly affect seed longevity include temperature, humidity, light exposure, and oxygen concentration [108]. Soil attributes, such as pH levels and mineral content, also have profound effects on seed survival within the soil seed bank [109,110]. Additionally, the seed microbiome, composed of endophytes and pathogens, subtly adjusts the seed microenvironment and defense mechanisms, thereby influencing seed longevity [111]. Our understanding of seed longevity in cultivated crops primarily stems from studies employing wet aging conditions to assess seed vigor. In contrast, a broader range of dry storage conditions has been applied to wild species. Despite this, the interaction between environmental factors and molecular regulators of longevity remains poorly understood.

### 3.1. Influence of Temperature on Seed Longevity

Storage temperature significantly impacts seed longevity by modulating enzymatic activities and metabolic processes within the seed [3]. Elevated temperatures, especially when combined with high moisture levels, intensify seed metabolism, accelerating aging and reducing longevity. Conversely, lower temperatures can alter the phenylpropanoid composition and permeability of the seed coat, as observed in Arabidopsis *transparent testa* mutants, which exhibit reduced longevity due to compromised seed coat integrity [96]. Therefore, precise temperature management is critical for optimizing seed longevity during storage.

Temperature-sensitive gene *DOG1* (DELAY OF GERMINATION 1), a key regulator of seed dormancy in Arabidopsis, also plays a significant role in seed longevity [41]. DOG1 promotes longevity by upregulating genes involved in stress responses, such as heat-shock proteins (*HSPs*) and late embryogenesis abundant proteins (*LEAs*). This regulation occurs partly through the activation of *ABI5* expression and in coordination with *ABI3* signaling [112]. Notably, DOG1 protein levels are influenced by seed maturation temperature, and loss of DOG1 function impairs seed dormancy induction at low maturation temperatures [113,114]. These findings suggest that DOG1 may orchestrate seed longevity responses to temperature through similar molecular mechanisms.

The effect of temperature on seed longevity varies significantly across species and genotypes, reflecting their ecological adaptations [14,109,115]. For example, warm temperatures during seed development generally enhance longevity in alpine species and Arabidopsis. In Arabidopsis, seeds matured at 23 °C exhibit greater longevity compared to those matured at 15 °C. However, in rice (*Oryza sativa*) and *Medicago truncatula*, elevated temperatures during development can reduce longevity, likely due to differences in their thermal tolerance and metabolic responses [3,116]. Conversely, low maturation temperatures (e.g., 10 °C) tend to diminish longevity in Arabidopsis but have little to no effect on *Medicago truncatula*, possibly due to species-specific adaptations to cooler climates [3].

Temperature profoundly influences seed longevity through its effects on enzymatic activity, seed coat properties, and stress response pathways. While warm temperatures may enhance longevity in some species (e.g., Arabidopsis and alpine plants), they can be detrimental to others (e.g., rice and soybean). Similarly, low temperatures may reduce longevity in certain species but have minimal effects in others. These variations highlight the need for species-specific strategies to optimize seed storage conditions and ensure long-term viability. For example, the relationship between temperature and seed longevity holds significant implications for the establishment and operation of seed gene banks. Seed gene banks play a pivotal role in the long-term preservation of plant genetic resources, safeguarding biodiversity and ensuring the availability of species for future generations. These facilities store seeds under controlled conditions, typically at temperatures of −18 °C to −20 °C and low relative humidity (15–20%), to minimize metabolic activity and extend seed longevity for decades or even centuries [3]. By maintaining seed viability over extended periods, gene banks serve as critical resources for crop improvement, ecological restoration, and the conservation of endangered species. However, for seeds that are sensitive to low temperatures, it is necessary to adjust storage conditions by slightly increasing the temperature to prevent a decline in seed viability. This tailored approach ensures the preservation of a wider range of species, including those with unique physiological requirements.

### 3.2. Effects of Water Availability During Seed Development on Seed Longevity

Water availability during seed development has a nuanced and context-dependent impact on seed longevity, varying according to species and the intensity of water stress [2]. Soybean seeds subjected to water stress during maturation exhibit the mature green seed phenotype, correlating with decreased longevity [117]. Notably, drought-induced reductions in *Medicago truncatula* seed longevity occur independently of visible chlorophyll retention [3], highlighting a distinct mechanism compared to other species where chlorophyll retention is a hallmark of decreased viability. However, in *Brassica rapa*, withholding irrigation during early seed-filling stages actually accelerates the accrual of seed longevity, enabling longer viability under dry storage [108]. Peanuts exemplify the species-specific response whereby drought stress during seed development can lead to increased longevity, which may be attributed to enhanced protein accumulation and subsequent efficient protein mobilization during germination [2,105]

The effect of water availability on seed longevity is complex and highly dependent on the developmental stage of the seed. Developing seeds exhibit a remarkable capacity to adapt their maturation processes in response to changes in water availability, thereby minimizing potential losses in longevity [2]. Field experiments on wheat that simulated varying rainfall patterns across different growth stages highlight the plasticity of seed development programs in adapting to water availability [118]. For instance, an increase in seed water content caused by wetting during development can reduce post-harvest longevity. However, if seeds are allowed to re-dry naturally, much of the lost longevity can be restored [119]. This demonstrates the intricate interplay between water availability, seed development, and longevity, emphasizing the importance of environmental conditions during seed maturation [118].

Although the exact molecular mechanisms behind water-regulated seed longevity are not fully understood, ABA signaling pathways appear to play a pivotal role in mediating seed maturation and dormancy under water stress [120]. Further exploration of these pathways is expected to shed light on the intricate regulatory networks that govern seed longevity in variable environmental contexts.

### 3.3. Light Exposure

Light exposure during seed development plays a critical role in shaping seed longevity, with its effects being mediated through photochemical reactions, chloroplast activity, and genetic regulation. During seed filling, embryos actively absorb 20–30% of incident light, particularly green and far-red wavelengths, which are essential for driving photosynthesis and energy production required for seed reserve accumulation [121]. Chloroplasts adapt their pigment composition and photosystem activity to optimize energy capture under varying light conditions, including in shade environments [108]. As seeds mature and transition into a desiccated state, chloroplasts disintegrate, and chlorophyll molecules undergo specialized degradation mechanisms that differ from those observed in senescent leaves [105,122]. This process is crucial for preventing the accumulation of reactive oxygen species (ROS) and other toxic compounds that could compromise seed longevity [123].

Photoperiod and light intensity during seed development both influence seed longevity, with evidence pointing to their regulatory roles in pathways related to longevity [15]. Light perception in *Arabidopsis* involves genes associated with the *DOG3* and *DOG6* loci, which are implicated in the genetic regulation of seed dormancy and longevity [120]. These findings suggest a genetic basis for light-mediated control of seed longevity, although the precise mechanisms remain to be fully elucidated. Future research should focus on cloning and characterizing key genes, such as those underlying the *DOG3* and *DOG6* loci, to better understand the molecular mechanisms linking light perception to seed longevity. Additionally, exploring the effects of light in a broader range of species, particularly crops, will provide valuable insights for the optimization of seed production and storage conditions to enhance longevity.

### 3.4. Nutrient Supply

Nutrient availability in the soil and the mother plant’s nutritional state profoundly influence seed yield and germination traits, reflecting a sophisticated interplay between genetics and environmental elements like temperature and light [14,105]. Various plant species, including tomato, *Arabidopsis*, and oilseed rape, provide evidence for the impact of nitrate, phosphate, and sulfate availability on seed traits [108]. Although the direct connection between nutrient availability and seed longevity has been less studied, new evidence suggests a potential link.

In barley, seeds harvested from plants grown under optimal nutrient conditions demonstrated enhanced longevity compared to those from nutrient-limited environments [105]. Nitrogen availability has been found to affect seed longevity in *Arabidopsis*, with higher nitrate levels corresponding to longer seed lifespans [124] and lower dormancy [125]. Changes in amino acid and glucoronate contents, along with alterations in gene transcripts linked to cell-wall metabolism, emphasize the impact of nutrient availability on seed composition and longevity [126]. Metabolic sensors like the Target of Rapamycin (TOR) complex and SnRK1 complex are crucial for integrating nutrient and energy signals to regulate seed development and longevity [127]. Mutant plants lacking these sensors exhibit decreased resistance to aging, highlighting the importance of metabolic sensing pathways in regulating seed longevity [105]. Thus, a deeper understanding of the intricate balance between nutrient availability, metabolic sensing, and seed longevity necessitates further investigation into the underlying molecular mechanisms.

### 3.5. Oxygen Levels

Oxygen levels during storage significantly affect seed longevity by modulating the formation of reactive oxygen species (ROS) and consequent oxidative damage to macromolecules [128]. Elevated oxygen levels induce DNA damage, correlating with increased chromosomal abnormalities and decreased seed viability and vigor [129,130]. Research on *Vicia faba* and soybean seeds stored under elevated oxygen pressures demonstrates the harmful effects of high oxygen levels, leading to rapid loss of germination capacity [117].

On the other hand, reduced oxygen levels can extend seed longevity during storage. ultra-dried Brassica seeds maintained viability for over three decades when kept in a modified atmosphere with lowered oxygen levels [131]. Vitamin E, recognized for its ability to scavenge lipid peroxy radicals, is believed to play a significant role in mitigating seed deterioration during storage in rice [132]. These results highlight the significance of oxygen regulation in maintaining seed viability during storage and point to the promise of modified atmospheric storage techniques for seed preservation.

## 4. Strategies to Enhance Seed Longevity

Current research highlights that seed longevity is determined not only by external environmental influences but also by intricate genetic factors [85]. The revelation of numerous genes deeply involved in seed longevity pathways raises the possibility of engineering seed varieties with enhanced longevity by targeting specific genetic elements. Additionally, considering that seeds often encounter suboptimal storage conditions that undermine their viability, exploring feasible methods to restore vigor to aged seeds is crucial for agriculture, laboratory research, and plant biodiversity conservation.

### 4.1. Extension of Seed Longevity Through Molecular Genetics

Genetic regulation plays a significant role in controlling seed longevity, offering a route to manipulate this trait through the alteration of gene expression using molecular genetics or genome editing technologies. Researchers have probed the molecular foundations of seed longevity, pinpointing essential genes (*SEED LONGEVITY-ASSOCIATED GENEs* (*SLAGs*)) and pathways. For example, genes involved in antioxidant defense systems, such as *SUPEROXIDE DISMUTASE* (*SOD*), *CATALASE* (*CAT*), and *PEROXIDASES* (*PODs*), help mitigate oxidative stress and preserve seed viability during storage [133]. Genes responsible for synthesizing and managing storage compounds, like LEAs and HSPs, have also been shown to significantly affect seed longevity [134].

With recent breakthroughs in genome editing, such as CRISPR-Cas9, scientists can now make precise adjustments to the genetic makeup of seeds. For example, editing the *FUSCA3* (*FUS3*) gene, which acts as a key regulator of seed maturation and longevity [45,135,136,137], has proven effective in enhancing seed storability and germination vigor in *Arabidopsis*. Similarly, CRISPR/Cas9-mediated knockout of the *LIPOXYGENASE 10* (*OsLOX10*) gene in rice led to increased seed longevity compared to the wild type under artificial aging conditions [138]. Knockout of type-II metacaspase (MCA-II) proteases via CRISPR in *Arabidopsis* disrupts proteostasis in seeds, thereby compromising seed longevity [75]. Leveraging molecular genetics and genome editing opens up the possibility of designing seeds with improved longevity characteristics tailored to specific environmental conditions or storage regimens. However, more research is needed to fully understand the complex genetic networks that govern seed longevity and to optimize the application of these techniques in crop improvement projects. Additionally, it is crucial to remember that gene editing can have unexpected consequences due to the complex nature of gene function. For example, although a knockout of the *RBOHD* gene can positively impact seed dormancy and longevity [46,100], it may also lead to undesirable phenotypes like a reduced growth rate and weakened immunity in *rbohd* mutants [139].

### 4.2. Extending Seed Longevity Through Seed Priming

Seed priming, a technique that partially activates germination processes without fully initiating germination, is known to improve seed performance and enhance stress tolerance in crop plants [140,141,142,143,144,145,146]. Priming typically involves treating seeds with water, osmotic solutions (e.g., polyethylene glycol), or specific bioactive compounds under controlled conditions. The seeds are then re-dried to their original moisture content, allowing them to remain dormant until favorable environmental conditions trigger germination [140,141,142,143,144,145,146]. This process aids in facilitating cellular repair mechanisms, which contributes to improved seedling vigor and crop yield [53]. Studies on leek (*Allium porrum*) and *Brassica oleracea* seeds have shown that priming is linked to heightened rates of DNA synthesis and accelerated cell division, leading to more rapid germination and quicker establishment of seedlings [147,148]. Moreover, priming induces changes in gene expression, such as the upregulation of DNA repair pathways and the increased activity of the protein repair enzyme L-ISOASPARTYL METHYLTRANSFERASE [149]. These molecular adjustments help reduce chromosomal abnormalities and enhance the overall quality of germination [53].

However, priming can occasionally diminish the storability or longevity of seeds. For example, the beneficial effects of seed priming were evident only for the first 15 days of storage at 25 °C in rice [150]. Beyond this period, the performance of the primed seeds declined, becoming even poorer than that of the non-primed seeds. The detrimental effects of storing the primed seeds at 25 °C were associated with impaired starch metabolism within the rice seeds [150,151]. To tackle this challenge, researchers developed an innovative priming method aimed at enhancing seed survival rates and maintaining seed longevity through the use of biologically active compounds, tested using *Arabidopsis* seeds [152]. Their findings indicated that priming with cell cycle inhibitors, such as mimosine, aphidicolin, hydroxyurea, and oryzalin, significantly improved both the survival rate and storability of seeds [152]. This suggests that the progression of the cell cycle during priming serves as a critical checkpoint affecting seed storability. By modulating this checkpoint through the inhibition of cell cycle progression, it may be possible to develop priming methods that preserve seed longevity while simultaneously boosting other aspects of seed performance.

### 4.3. Revitalizing Old Seeds

Despite the importance of optimal storage, real-world constraints such as infrastructure limitations, natural disasters, and logistical issues can hinder the maintenance of ideal seed storage environments. This is especially true for rare or experimentally conserved germplasms that may have been subjected to subpar storage conditions, reducing their viability. Therefore, developing methods to revive even a portion of these seeds carries immense value.

Aged or poorly stored seeds often suffer from energy depletion, reduced enzyme activities, and hypoxic conditions during germination [105,153], necessitating targeted interventions to restore vital components. Hydrogen peroxide, for instance, has been successfully used to resuscitate four-year-old squash (*Cucurbita pepo*) seeds by acting as an oxygen supplement [154]. Beyond direct chemical treatments, in vitro tissue culture techniques present a promising avenue to rescue the germination potential of immature and aged cucurbit seeds. This method extracts embryos from deteriorating seeds and cultivates them in a nutrient-rich, sterile environment conducive to germination, with success partially hinging on the selection, concentration, and synergistic combination of specific plant growth regulators. Ethylene supplementation, for example, has been shown to expedite germination in aged *Brassica napus* seeds [155], while cucumber seed regeneration has been effectively achieved with the combined use of 1-naphthaleneacetic acid and 6-benzylaminopurine [156,157]. Low doses of epibrassinolide have also been found to improve germination rates in pepper (*Capsicum annuum*) seeds [158]. GA3 holds potential for enhancing seed germination when applied externally, though its effectiveness is highly dependent on dosage—lower concentrations can stimulate germination, while higher amounts can inhibit it [159,160]. Interestingly, alternative gibberellin compounds like GA4/7 may outperform GA3 in promoting cucurbit seed germination, suggesting potential benefits over conventional GA3 usage [159]. Notably, Zn-specific chelator TPEN (N, N, N’, N’-Tetrakis (2-pyridylmethyl) ethylenediamine) can significantly delay the aging process of the seeds by regulating the levels of glutathione [161], suggesting that free metal ions released due to the loss of membrane integrity may be both a consequence of and a contributing factor to seed aging.

While rescue strategies play a vital role in rejuvenating aged seeds, the fundamental importance of proper seed storage cannot be underestimated. To optimally preserve laboratory seeds, meticulous attention must be paid to keeping seed density low in centrifuge tubes, using hermetically sealed containers for cold storage, and maintaining ideal relative humidity levels to prevent moisture damage [7]. When it comes to preserving laboratory seeds, optimal storage necessitates meticulous attention to several key aspects: first, ensuring minimal seed density within centrifuge tubes, thereby minimizing the risk of accelerated deterioration; second, employing hermetically sealed containers for long-term preservation in cold environments (−20 ± 4 °C); maintaining the relative humidity level (15 ± 3%) to prevent moisture-induced damage; and incorporating silica gel pellets to act as a desiccant, absorbing excess moisture and prolonging seed lifespan and viability [7]. Moreover, the strategic integration of silica gel pellets into the storage system serves as an effective desiccant measure, actively absorbing excess moisture and thereby extending the lifespan and viability of the seeds. By combining stringent storage practices with these revitalization techniques, researchers can optimize the successful germination of aged seeds by mitigating the multiple factors that contribute to seed deterioration and loss of vigor during storage.

## 5. Techniques for Assessing Seed Longevity

The study of seed vigor decay during storage necessitates robust assessment methods, with Accelerated Aging (AA) and Controlled Deterioration (CD) tests being pivotal in predicting seed longevity [162,163]. These tests simulate the natural aging process under controlled settings yet accelerate it to facilitate practical experimentation [8,163]. Seed industry professionals heavily rely on these evaluations to determine seed vigor and shelf life. In AA tests, seeds are exposed to heightened temperatures and 100% relative humidity, while in CD tests, seeds are enclosed in aluminum foil packets under conditions of high moisture and temperature [162]. Although both methods aim to predict seed longevity, the different aging environments—from the aerobic conditions of AA to the anaerobic or low-oxygen conditions of CD—produce varying aging kinetics, thereby influencing the way seeds deteriorate over time [164].

A wide array of techniques is used to measure seed longevity, starting with the standard germination test [163,165]. This test assesses seed viability by measuring the proportion of germinated seeds, providing a simple pass/fail outcome. However, it might not detect subtle, non-lethal degradation that occurs during the early stages of storage [163,166]. As storage conditions progressively impair seeds, mortality rates rise, typically following an S-shaped pattern. Another method, the TTC (triphenyl tetrazolium chloride) staining assay, quickly gauges viability based on dehydrogenase activity within seeds, turning viable embryos red as a qualitative or semi-quantitative indicator of seed health [166,167]. Though convenient for preliminary checks, TTC does not provide detailed quantification beyond distinguishing live from dead seeds.

Advances in technology, such as high-throughput scanning, permit quantitative evaluation of seed viability, although embryo dissection may be required, which can present operational challenges. More recently, RNA integrity analysis has emerged as a potent means to monitor seed deterioration during dry storage [165,168]. RNA stability tightly correlates with seed endurance, as fragmented RNA signifies declining viability. Researchers can examine the extent of RNA degradation by performing electrophoresis on total RNA and calculating the RNA Integrity Number (RIN) [165,168]. RIN values, ranging from one (completely degraded RNA) to ten (intact RNA), serve as a strong predictor of seed longevity. The association between RIN scores and germination potential highlights the critical role of embryonic RNA integrity in maintaining seed viability [165]. Collectively, these diverse methodologies provide profound insights into the intricate processes governing seed longevity, empowering researchers to develop more informed conservation strategies and breed seeds with greater resilience and optimized agricultural sustainability.

## 6. Challenges, Questions, and Approaches

While advancements in biotechnology and molecular biology have led to significant insights into the mechanisms governing seed vigor and longevity, numerous challenges persist in translating these discoveries into practical applications. One of the primary obstacles in enhancing seed vigor and longevity is the complexity of the underlying biological processes. These processes involve intricate networks of genetic, metabolic, and environmental interactions that are not yet fully understood [14]. For instance, the role of ROS in seed aging is well documented, but the precise mechanisms by which ROS damage cellular components and the ways to mitigate this damage remain areas of ongoing research [63]. Another challenge is the variability among different plant species and genotypes. Seeds of various species exhibit different sensitivities to environmental stressors and storage conditions, making it difficult to develop universal strategies for improving seed quality [169]. Additionally, there is a need for a more comprehensive understanding of how abiotic stresses, such as temperature and humidity, interact with seed physiology to affect longevity [105]. Moreover, economic constraints and the lack of standardized protocols for seed testing and evaluation pose significant barriers to progress [163]. There is a continuous need for cost-effective and reliable methods to assess seed quality, which can be applied globally, from small-scale farmers to large agribusinesses [162,170].

To address these challenges, an interdisciplinary approach is required, combining genetics, biochemistry, and agronomy with biotechnology and precision agriculture [14,171]. Strategies could include the use of genetic engineering to introduce or enhance protective mechanisms against ROS, the development of species-specific storage guidelines based on detailed physiological studies, and the creation of robust seed quality assessment tools [170]. Furthermore, global collaboration, resource and knowledge sharing, and interdisciplinary approaches could significantly accelerate the discovery of effective solutions. For instance, applying statistical methods from mathematics to seed longevity research, such as using probit analysis in R to model germination data for stored seeds—whether derived from designed experiments or collected as part of routine viability monitoring in seed gene banks—can provide valuable insights [172]. This approach will advance our understanding of seed longevity across different taxa in response to varying harvesting and post-harvest treatments and under diverse storage environments. Such advancements will contribute to broader efforts in conserving agricultural biodiversity while also enhancing our knowledge of seed physiological quality and the factors influencing the natural capital value of seeds.

While enhancing seed longevity offers clear benefits, such as improved storage stability and extended viability, it is essential to consider the potential downsides. One significant concern is the possible trade-off between seed longevity and germination vigor. Seeds engineered for extended shelf life might exhibit reduced field performance due to alterations in metabolic pathways that affect germination efficiency and early seedling establishment [2]. Additionally, increasing seed longevity could inadvertently select for traits that delay germination, potentially leading to uneven crop stands and reduced uniformity in planting populations, which are detrimental to crop management and yield consistency [15,85]. Therefore, when constructing crops through overexpression or gene editing of *SLAG*s, this issue should be taken into account.

Furthermore, the genetic modifications required to achieve enhanced longevity might have unintended consequences for plant health and susceptibility to diseases. For instance, changes in seed composition could impact the plant’s natural defense mechanisms against pathogens and pests [173]. Lastly, there is an ecological consideration; seeds with extended viability might persist in the soil longer, potentially outcompeting native species and disrupting local ecosystems if they are not properly managed [174].

## Figures and Tables

**Table 1:** Genes involved in regulating seed longevity in *Arabidopsis*.

Locus	Gene	Effect	Pathway	Reference (PubMed ID)
AT4G13250	NYC1	positive	Chlorophyll degradation	22751379
AT3G48190	ATM	negative	DNA repair	27503884
AT5G40820	ATR	negative	DNA repair	27503884
AT3G05210	ERCC1	positive	DNA repair	35858436
AT1G16970	KU70	positive	DNA repair	35858436
AT5G57160	LIG4	positive	DNA repair	20584150
AT1G66730	LIG6	positive	DNA repair	20584150
AT1G25580	SOG1	negative	DNA repair	35858436
AT1G21710	OGG1	positive	DNA repair	22473985
AT2G31320	PARP1	positive	DNA repair	35858436
AT5G22470	PARP3	positive	DNA repair	24533577
AT1G14410	WHY1	positive	DNA repair	37351567
AT2G02740	WHY3	positive	DNA repair	37351567
AT5G64520	XRCC2	positive	DNA repair	35858436
AT1G34790	TT1	positive	Flavonoid biosynthesis	10677433
AT5G48100	TT10	positive	Flavonoid biosynthesis	10677433
AT5G42800	TT3	positive	Flavonoid biosynthesis	10677433
AT3G55120	TT5	positive	Flavonoid biosynthesis	10677433
AT5G07990	TT7	positive	Flavonoid biosynthesis	10677433
AT4G09820	TT8	positive	Flavonoid biosynthesis	10677433
AT3G28430	TT9	positive	Flavonoid biosynthesis	10677433
AT3G24650	ABI3	positive	Hormone, ABA	12231895
AT3G18490	ASPG1	positive	Hormone, ABA	29648652
AT5G45830	DOG1	positive	Hormone, ABA	17065317
AT2G36610	ATHB22	positive	Hormone, GA	24335333
AT5G65410	ATHB25	positive	Hormone, GA	24335333
AT1G14440	ATHB31	positive	Hormone, GA	24335333
AT1G80340	GA3OX2	positive	Hormone, GA	24335333
AT2G01570	RGA1	negative	Hormone, GA	24335333
AT1G14920	RGA2	negative	Hormone, GA	24335333
AT1G66350	RGL1	negative	Hormone, GA	24335333
AT3G03450	RGL2	negative	Hormone, GA	24335333
AT5G17490	RGL3	negative	Hormone, GA	24335333
AT1G09570	PHYA	negative	Light	27227784
AT2G18790	PHYB	negative	Light	27227784
AT2G45970	CYP86A8	positive	Lipid biosynthesis	32519347
AT3G47860	AtCHL	positive	Lipid peroxidation	23837879
AT5G58070	AtTIL	positive	Lipid peroxidation	23837879
AT1G55020	LOX1	negative	Lipid peroxidation	28371855
AT1G28440	AtHSL1	positive	LRR-RLK	35763091
AT2G27500	BG14	positive	Metabolism Carbohydrate	36625794
AT2G47180	GOLS1	positive	Metabolism Galactose	26993241
AT1G56600	GOLS2	positive	Metabolism Galactose	26993241
AT1G30370	AtDLAH	positive	Metabolism Lipid	21856645
AT2G19900	NADP-ME	positive	Metabolism Malate	29744896
AT4G15940	AtFAHD1a	negative	Metabolism Oxoacid	33804275
AT4G02770	PSAD1	positive	PHOTOSYSTEM	32519347
AT1G62710	β-VPE	positive	Protein catabolism	30782971
AT2G26130	RSL1	positive	Protein degradation	24388521
AT5G45360	SKIP31	positive	Protein degradation	37462265
AT5G53000	TAP46	positive	Protein dephosphorylation	25399018
AT3G25230	ROF1	positive	Protein isomerization	22268595
AT5G48570	ROF2	positive	Protein isomerization	22268595
AT3G48330	PIMT1	positive	Protein repair	19011119
AT3G57520	AtSIP2	negative	Raffinose catabolism	34553917
AT4G02750	SSTPR	positive	RNA modification	32519347
AT1G19570	DHAR1	positive	ROS detoxification	32519347
AT1G05250	PRX2	positive	ROS detoxification	31600827
AT2G41480	PRX25	positive	ROS detoxification	31600827
AT5G64120	PRX71	positive	ROS detoxification	31600827
AT5G47910	RBOHD	negative	ROS production	32519347
AT1G19230	RBOHE	negative	ROS production	32519347
AT1G64060	RBOHF	negative	ROS production	32519347
AT3G17520	LEA	positive	Seed development	32519347
AT5G44120	CRUA	positive	Seed storage protein	26184996
AT1G03880	CRUB	positive	Seed storage protein	26184996
AT4G28520	CRUC	positive	Seed storage protein	26184996
AT4G36920	AtAP2	positive	TF AP2/EREBP	10677433
AT5G53210	SPCH1	positive	TF bHLH	32519347
AT2G34140	CDF4	positive	TF DOF	27227784
AT1G29160	COG1	positive	TF DOF	31600827
AT4G00940	DOF4.1	negative	TF DOF	35845633
AT5G42630	ATS	positive	TF G2-LIKE	10677433
AT1G79840	GL2	positive	TF HB	10677433
AT1G62990	KNAT7	negative	TF HB	32519347
AT5G54070	AtHSFA9	positive	TF HSF	32683703
AT5G15800	AGL2	negative	TF MADS	32519347
AT1G18710	MYB47	positive	TF MYB	32519347
AT1G21970	LEC1	positive	TF NF-YB	19754639
AT2G38470	WRKY33	positive	TF WRKY	26410298
AT4G32770	VTE1	positive	Tocopherol biosynthesis	15155886
AT2G18950	VTE2	positive	Tocopherol biosynthesis	15155886
AT1G73190	TIP3.1	positive	Transmembrane transport	26019256
AT1G17810	TIP3.2	positive	Transmembrane transport	26019256

## Data Availability

All the data used in this review paper are available online.

## Abbreviations

The following abbreviations are used in this manuscript:

SLAG	SEED LONGEVITY-ASSOCIATED GENE
ABI3	ABSCISIC ACID-INSENSITIVE3
TIP3;1	TONOPLAST INTRINSIC PROTEIN 3;1
ROF1	ROTAMASE FKBP 1
DREB2	DROUGHT RESPONSIVE ELEMENT BINDING FACTOR 2
IAA27	AUXIN-RESPONSIVE PROTEIN 27
DOG1	DELAY OF GERMINATION 1
COG1	COGWHEEL1
CDF4	CYCLING DOF FACTOR 4
PER1A	PEROXIDASE 1A
ATM	ATAXIA TELANGIECTASIA MUTATED
ATR	ATM AND RAD3-RELATED
SOG1	SUPPRESSOR OF GAMMA 1
LIG4	DNA LIGASE 4
XRCC2	X-RAY REPAIR CROSS COMPLEMENTING 2
PARP1	POLY(ADP-RIBOSE) POLYMERASE 1
ERCC1	EXCISION REPAIR CROSS COMPLEMENT-ING-GROUP 1
8-OXOG	8-OXOGUANINE
DSBs	DOUBLE-STRAND BREAKS
HR	HOMOLOGOUS RECOMBINATION
ROS	REACTIVE OXYGEN SPECIES
SnRK2	SUCROSE NON-FERMENTING 1-RELATED PROTEIN KINASE 2
ABA	ABSCISIC ACID
EM1	EARLY METHIONINE 1
AtHB25	ARABIDOPSIS THALIANA HOMEOBOX 25
TOR	TARGET OF RAPAMYCIN
SOD	SUPEROXIDE DISMUTASE
CAT	CATALASE
POD	PEROXIDASES
